# Optical coherence tomography angiography findings in patients affected by giant cell arteritis, with and without ocular involvement: a pilot study

**DOI:** 10.3389/fmed.2024.1408821

**Published:** 2024-08-12

**Authors:** Lorenzo Vannozzi, Cristina Nicolosi, Giulio Vicini, Daniela Bacherini, Dario Giattini, Maria Letizia Urban, Adalgisa Palermo, Danilo Malandrino, Federica Bello, Gianni Virgili, Fabrizio Giansanti

**Affiliations:** ^1^Eye Clinic, Neuromuscular and Sense Organs Department, Careggi University Hospital, Florence, Italy; ^2^Department of Neurosciences, Psychology, Drug Research and Child Health, University of Florence, Florence, Italy; ^3^Azienda USL Toscana Nord Ovest, Pisa, Italy; ^4^Department of Experimental and Clinical Medicine, University of Florence, Florence, Italy

**Keywords:** giant cell arteritis, OCTA, optical coherence tomography angiography, Horton arteritis, OCT

## Abstract

**Purpose:**

We evaluated the clinical features and retinal and disk perfusion characteristics by using optical coherence tomography (OCT) and optical coherence tomography angiography (OCTA) in a subset of giant cell arteritis (GCA) patients who manifested anterior ischemic optic neuropathy (AION), in a subset of GCA patients without ocular involvement, and in a control group composed of healthy controls.

**Methods:**

We performed an observational study on the eyes of GCA patients affected by arteritic AION both in acute and chronic phases, unaffected eyes of AION, eyes of GCA patients without ocular involvement, and in a control group of healthy eyes of healthy individuals. All patients underwent a complete ophthalmic examination and an OCT and OCTA of the macula and the disk.

**Results:**

The study evaluated 10 eyes of GCA patients with AION (AION group), 8 unaffected eyes of GCA patients with AION in another eye (unaffected eyes of AION group), 16 eyes of GCA patients without ocular involvement (non-ocular group), and 22 eyes of healthy patients (healthy group). The ganglion cell complex (GCC) superior and inferior thicknesses were significantly lower in the AION group compared to the unaffected eyes of the AION group (*p* = 0.045 and *p* = 0.034, respectively). All OCTA vascular density parameters of the optic disk analyzed in this study (optic nerve head (ONH) whole, superior, inferior, radial peripapillary capillary plexus (RPCP) whole, superior, inferior, lamina cribrosa (LC) whole, superior, inferior) resulted significantly lower in the AION group compared to the unaffected eyes group (*p* < 0.05 for all the comparisons). The ONH whole and inferior were statistically higher in the healthy group in comparison to the group of GCA patients without ocular involvement (*p* = 0.008 and *p* = 0.006, respectively). The ONH inferior was also statistically higher in the unaffected eyes of the AION group in comparison to the non-ocular group (*p* = 0.045). Regarding the OCTA macular vessel density parameters, the superficial capillary plexus (SCP), whole and inner, were statistically lower in the AION group compared with the unaffected eyes of the AION group.

**Conclusion:**

We found a profound vascular impairment in eyes affected by AION and areas of hypoperfusion in the eyes of patients with GCA without ocular involvement, good BCVA, and no clinically significant features. We hypothesized that these areas of lower vessel density might represent areas of subclinical hypoperfusion that cannot be detected ophthalmoscopically.

## Introduction

GCA, formerly known as temporal arteritis, is the most common form of systemic vasculitis in patients aged ≥50 years. GCA typically affects large and medium-sized vessels characterized by granulomatous inflammation, which usually involves the cranial branches of the arteries originating from the aortic arch (i.e., the external carotid branches of the occipital and temporal arteries, ophthalmic, vertebral, distal subclavian, and axillary arteries) (1–4). Common presenting features of GCA include headache, constitutional symptoms, jaw claudication, scalp tenderness, visual disturbances, and sudden vision loss (5). GCA is closely related to polymyalgia rheumatica (PMR), which is an inflammatory condition of unknown cause characterized by aching and morning stiffness in the cervical region and shoulder and pelvic girdles (1). GCA is the most common systemic vasculitis worldwide, with a prevalence rate of 87.9 to 250 per 100,000 individuals (6). The incidence typically peaks during the seventh and eighth decades of life. Women account for 65–75% of patients (7). Some experts consider them to be different phases of the same disease that affect people of middle age and older and frequently occur together (7).

GCA affects the ocular circulation in more than half of cases and can cause irreversible bilateral blindness if not rapidly recognized and treated, representing one of the few true ophthalmic emergencies (8).

The most common GCA mechanism that leads to irreversible visual loss is AION, resulting from the involvement of the short posterior ciliary arteries supplying the ONH (9). The recently introduced non-invasive OCTA technique can provide high-resolution vascular images of the retina and the ONH in a non-invasive way, and its use in the analysis of vascular impairment occurring in GCA patients has been described only in a few studies (10–13).

This study aimed to evaluate the clinical features and subtle retinal and disk perfusion characteristics, by using OCT and OCTA in a subset of GCA patients who manifested AION, in a subset of GCA patients without ocular involvement, and in a control group composed of healthy controls.

## Materials and methods

We performed an observational study on 10 eyes of 9 GCA patients affected by arteritic AION both in acute and chronic phases (AION GCA patients’ group), 8 unaffected eyes of AION group patients (unaffected eyes AION GCA patients’ group), 16 eyes of 8 GCA patients without ocular involvement (non-ocular GCA patients’ group), and a control group of 22 healthy eyes of 22 healthy individuals recruited as volunteers (healthy controls’ group). Patients from the three groups were matched for sex and age. The GCA patients were recruited through the eye clinic and the Department of Experimental and Clinical Medicine, Careggi University Hospital, Florence, Italy. The study was approved by the Careggi University Hospital Research Ethics Board, adhered to the tenets of the Declaration of Helsinki, and was conducted from January 2020 to December 2021. Inclusion criteria for the AION group were the GCA diagnosis and acute or chronic AION ocular involvement; for the non-ocular group, the GCA diagnosis and the absence of any signs and symptoms characteristic of ischemic ocular involvement.

The GCA diagnosis was made according to the criteria used in the GIACTA trial (14), listed as follows:

- Age ≥ 50 years.
- Westergren ESR > 30 mm/h or CRP ≥ 1 mg/dL (including a presenting history of ESR > 50 mm/h).
- And at least one of the following:
  o Unequivocal cranial symptoms of GCA (new-onset localized headache, scalp or temporal artery tenderness, ischemia-related vision loss, or otherwise unexplained mouth or jaw pain upon mastication).
  o Symptoms of PMR, defined as shoulder and/or hip girdle pain associated with inflammatory morning stiffness.
- And at least one of the following:
  o Temporal artery biopsy reveals features of GCA.
  o Evidence of large-vessel vasculitis by angiography or cross-sectional imaging studies such as MRA, CTA, or PET-CTA.

All patients included in the study underwent a complete ophthalmic examination, including best-corrected visual acuity (BCVA) measurement using decimal Snellen charts, intraocular pressure evaluation, slit lamp examination of the anterior segment, dilated fundus examination, B-scan OCT, and OCTA. Patients with high refractive errors (myopia greater than 6 D, hypermetropia, or astigmatism greater than 3 D), relevant ocular pathologies other than GCA-related alterations, or media opacities that precluded good visualization of the fundus were excluded.

OCT and OCTA examinations were performed by experienced clinicians (C.N. and D.B.) under full pharmacologic mydriasis with topical tropicamide 1% using the RS-3000 Advance 2 OCT (NIDEK Co. Ltd., Gamagori, Japan). OCTA RS-3000 Advance 2 uses an 880-nm wavelength with a scanning speed of 85,000A scans/s and provides high-quality images that allow the qualitative and quantitative assessment required for our evaluation.

**Figure 1:**
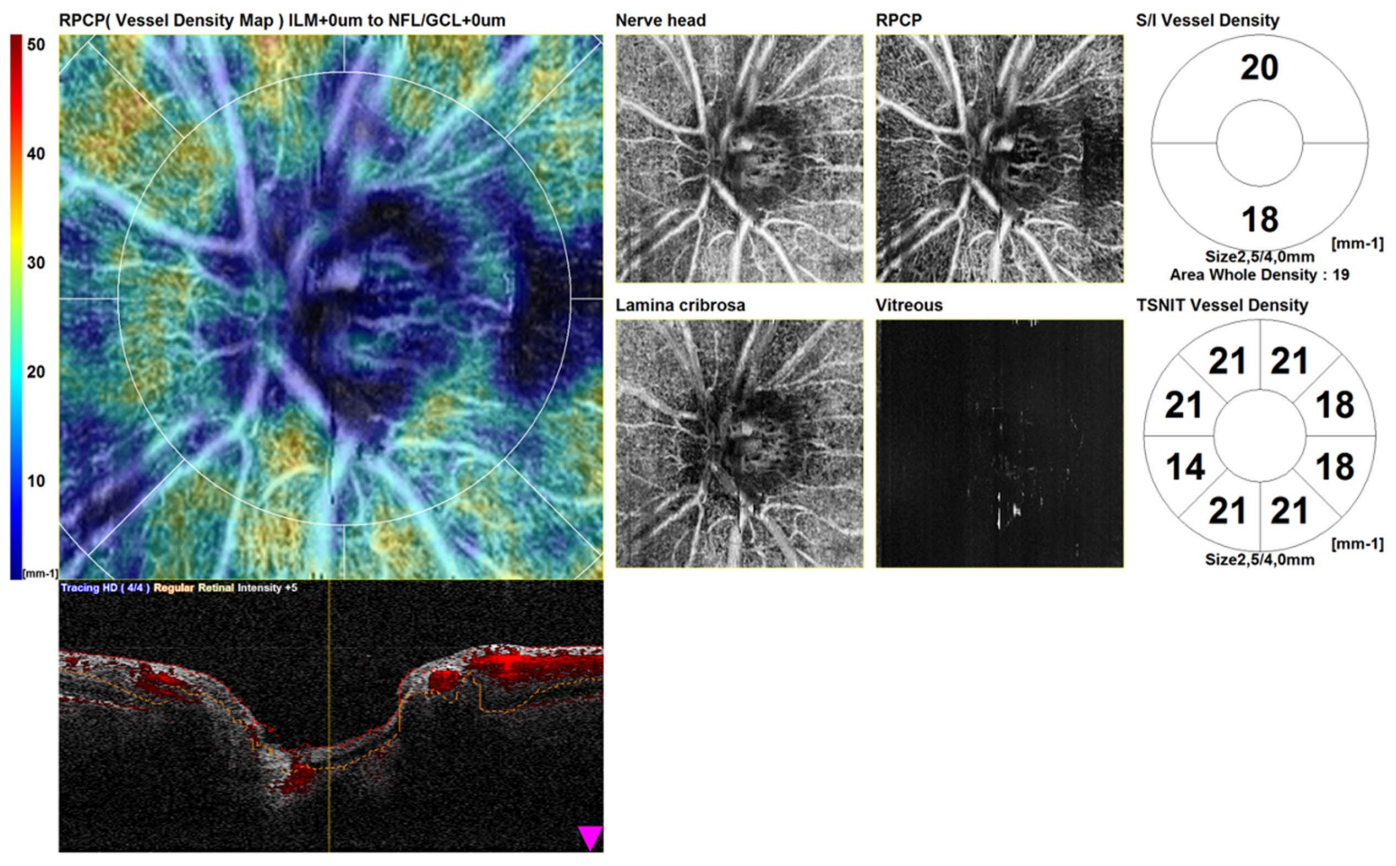
Optical coherence tomography angiography disk map (3 mm × 3 mm) of a healthy patient.

The scans consisted of a structural OCT disk circle (3.45 mm), a structural OCT macula map (3 mm × 3 mm), an OCTA disk map (3 mm × 3 mm) centered on the optic disk, and an OCTA (3 mm × 3 mm) centered on the fovea. Automated segmentation of OCTA images with manual adjustment was performed. The tracing HD plus function of the RS-3000 Advance 2 OCT system reduces motion and blink artifacts. We included in the study only high-quality scans. We excluded poor-quality scans and scans with incorrect segmentation or motion artifacts. Scans with a signal strength index of <7/10 were repeated. We conducted a qualitative and quantitative assessment. Figure 1 shows OCTA disk scans in a healthy patient.

Updated Advance 2 OCTA AngioScan software was used to evaluate structural OCT and OCTA images. Central retinal thickness (CRT) and significance maps for the GCC and retinal nerve fiber layer (RNFL) were generated from structural OCT scans by using in-built software, and vessel densities were analyzed on OCTA scans.

Advance 2 OCTA AngioScan software was used to evaluate the vessel densities. For each eye included in the study, we performed a quantitative evaluation. Vessel density was defined as the percentage of the sample area occupied by vessel lumens. The vessel density was quantified in the OCTA disk at the ONH, RPCP, and LC levels. In particular, the disk vessel density on OCTA scans was analyzed at the following levels: ONH whole, ONH superior, ONH inferior, RPCP whole, RPCP superior, RPCP inferior, LC whole, LC superior, and LC inferior. The macular vessel densities were evaluated at the SCP, deep capillary plexus (DCP), choriocapillaris (CC), and choroid. In particular, the macular vessel density was analyzed at the following levels: SCP whole, SCP inner, SCP outer, DCP whole, DCP inner, DCP outer, outer retina to choriocapillaris (ORCC) whole, ORCC inner, ORCC outer, CC whole, CC inner, CC outer, choroid whole, choroid inner, and choroid outer. Images were reviewed by two investigators (C.N. and D.B.) for segmentation accuracy.

We made comparisons of OCT and OCTA parameters between the groups: AION-affected eyes were compared to unaffected eyes of the same GCA patients (AION group vs. unaffected eyes group), eyes of GCA patients without ocular involvement were compared to eyes of normal patients (non-ocular group vs. healthy group), and eyes of GCA patients without ocular involvement were compared to the unaffected eyes of GCA patients with AION in the other eye (non-ocular group vs. unaffected eyes group).

We also performed a qualitative morphological assessment of optic disk perfusion characteristics based on the analysis of color-coded types of vessel density maps, automatically elaborated using the AngioScan software.

Statistical analysis was performed using SPSS Statistics (SPSS Inc., Chicago, IL, United States) software for Mac (Version 26.0). Between-group comparisons in OCT and OCTA quantitative parameters were performed using a two-tailed Student’s *t*-test with 95% confidence intervals. Statistical significance was defined as a *p*-value of <0.05.

## Results

### Demographic and clinical characteristics

The study evaluated 58 eyes from different groups: 10 eyes of 9 GCA patients with AION (3 males, 6 females, mean age 78.7 ± 7.1 years) (AION group); 8 unaffected eyes of 8 GCA patients with AION in another eye (unaffected eyes of AION group); 16 eyes of 8 GCA patients without ocular involvement (3 males, 5 females, mean age 78.4 ± 1.2 years) (non-ocular group); and 22 eyes of 11 healthy patients (4 males, 7 females, mean age 78.6 ± 4.5 years) (healthy group). In the AION group, only one patient had bilateral involvement; the other 8 patients had monoliteral involvement.

In GCA patients of the AION group, AION was the onset manifestation that led to the diagnosis of the disease. In the AION group, one patient had bilateral AION involvement. AION eyes in the acute phase ophthalmoscopically showed ONH swelling and pallor, and AION eyes in the late stage showed optic atrophy with generalized optic disk pallor. Unaffected eyes showed unremarkable changes.

The lowest BCVA in the AION group was hand motion, the highest BCVA was 3.2/10, and in eccentric fixation, the median BCVA was 1/10. In the unaffected eyes of the AION group, the lowest BCVA was 8/10, the highest BCVA was 10/10, and the median BCVA was 9/10. In the non-ocular group, the lowest BCVA was 8/10, the highest BCVA was 10/10, and the median BCVA was 10/10. In the healthy group, BCVA was 10/10 in all patients.

In the AION group, four affected eyes of four patients were evaluated with OCT and OCTA in the acute phase of AION presentation (within 4 days from AION onset) and six affected eyes of 5 patients were evaluated in the post-acute phase (from 3 months to 4 years from AION onset, median time 1 year).

### Optical coherence tomography and optical coherence tomography angiography results

**Figure 2:**
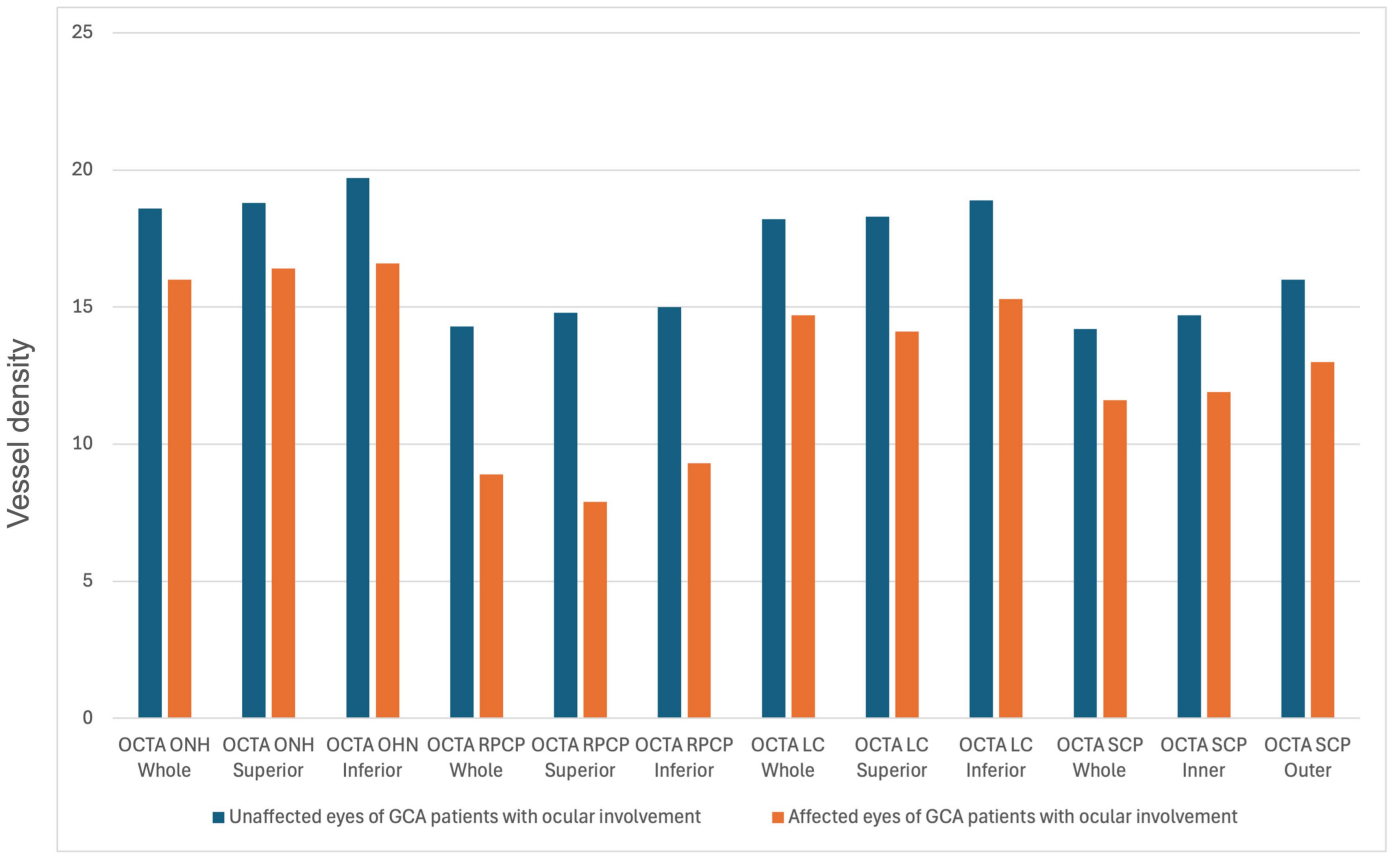
Statistically significant differences (*p*-value <0.05) in the comparison of optical coherence tomography angiography (OCTA) parameters between unaffected and affected eyes of giant cell arteritis patients with ocular involvement. ONH, Optic nerve head; RPCP, radial peripapillary capillary plexus; LC, lamina cribrosa; SCP, superficial capillary plexus.

The comparisons of structural OCT and OCTA parameters between the different groups are shown in Table 1. Regarding structural OCT data, the CRT difference was not significantly different between the groups. The GCC superior and inferior thicknesses were significantly lower in the AION group compared to the unaffected eyes of the AION group (differences: −11.5 [*p* = 0.045] and −13.9 [*p* = 0.034], respectively). The inferior RNFL thickness was significantly higher in normal patients compared to GCA patients without ocular involvement (non-ocular group) (difference: +16.5, *p* = 0.04), while the nasal RNFL thickness was higher in the unaffected eyes of the AION group compared to the non-ocular group (difference: +23.7, *p* = 0.011). OCTA vascular density parameters of the optic disk analyzed in this study (ONH whole, ONH superior, ONH inferior, RPCP whole, RPCP superior, RPCP inferior, LC whole, LC superior, LC inferior) resulted significantly lower in the AION group compared to the unaffected eyes group (*p* < 0.05 for all the comparisons) (Figure 2). The ONH whole and inferior were statistically higher in the healthy group in comparison to the non-ocular group (difference: +1.7 [*p* = 0.008] and + 1.7 [*p* = 0.006], respectively). The ONH inferior was also statistically higher in the unaffected eyes of the AION group in comparison to the non-ocular group (difference: +2.0, *p* = 0.045).

**Table 1:** Comparison of optical coherence tomography (OCT) and optical coherence tomography angiography (OCTA) parameters of giant cell arteritis (GCA) patients without ocular involvement (A), vs. normal patients (B), vs. GCA patients with ocular involvement and unaffected eyes (C), and comparison between unaffected vs. affected eyes in GCA patients (D).

OCT/OCTA variable	A – GCA patients without ocular involvement (mean value)	B – Normal patients (mean value [difference vs. A])	C – GCA patients with ocular involvement: unaffected eye (mean value [difference vs. A])	D – GCA patients with ocular involvement: affected eye (mean value [difference vs. C])
**Structural OCT**				
CRT	265.2	270.3 [+5.1]	268.2 [+3.0]	259.3 [−8.9]
GCC superior	87.2	92 [+4.8]	86.6 [−0.6]	75.1 [−11.5, *p* = 0.045]*
GCC inferior	89.2	94.9 [+5.7]	89.8 [+0.6]	75.9 [−13.9, *p* = 0.034]*
RNFL temporal	66.3	67.5 [+1.2]	54.1 [−12.2]	80.2 [+26.1]
RNFL superior	117.8	128.3 [+10.5]	111.3 [−6.5]	132.5 [+21.2]
RNFL nasal	74.0	77.7 [+3.7]	97.7 [+23.7, *p* = 0.011]*	75.6 [−22.1]
RNFL inferior	110.6	127.1 [+16.5, *p* = 0.04]*	121.4 [+10.8]	134.4 [+13.0]
**OCTA disk**				
ONH whole	17.4	19.1 [+1.7, *p* = 0.008]*	18.6 [+1.2]	16 [−2.6, *p* = 0.013]*
ONH superior	18.5	19.7 [+1.2]	18.8 [+0.3]	16.4 [−2.4, *p* = 0.03]*
OHN inferior	17.7	19.4 [+1.7, *p* = 0.006]*	19.7 [+2.0, *p* = 0.045]*	16.6 [−3.1, *p* = 0.021]*
RPCP whole	15.9	17 [+1.1]	14.3 [−1.6]	8.9 [−5.4, *p* = 0.026]*
RPCP superior	16.7	17.7 [+1.0]	14.8 [−1.9]	7.9 [−6.9, *p* = 0.016]*
RPCP inferior	15.9	17.4 [+1.5]	15.0 [−0.9]	9.3 [−5.7, *p* = 0.017]*
LC whole	17.9	18.4 [+0.5]	18.2 [+0.3]	14.7 [−3.5, *p* = 0.02]*
LC superior	18.4	18.1 [−0.3]	18.3 [−0.1]	14.1 [−4.2, *p* = 0.018]*
LC inferior	18.3	19.4 [+1.1]	18.9 [+0.6]	15.3 [−3.6, *p* = 0.03]*
**OCTA macula**				
SCP whole	15.2	16.9 [+1.7]	14.2 [−1.0]	11.6 [−2.6, *p* = 0.041]*
SCP inner	15.9	17.4 [+1.5]	14.7 [−1.2]	11.9 [−2.8, *p* = 0.036]*
SCP outer	16.6	18.3 [+1.7]	16.0 [−0.6]	13.0 [−3.0, *p* = 0.043]*
DCP whole	11.6	14.8 [+3.2]	12.3 [+0.7]	9.4 [−2.9]
DCP inner	12.1	15.2 [+3.1]	12.4 [+0.3]	9.5 [−2.9]
DCP outer	13	16.3 [+3.3]	13.5 [+0.5]	10.5 [−3.0]
ORCC whole	18.7	19.7 [+1.0]	19.3 [+0.6]	17.1 [−2.2]
ORCC inner	18.7	19.6 [+0.9]	19.3 [+0.6]	16.9 [−2.4]
ORCC outer	19.1	20.4 [+1.3]	20.4 [+1.3]	18.2 [−2.2]
CC whole	14.6	16.3 [+1.7]	17.1 [+2.5]	14.2 [−2.9]
CC inner	14.8	16.1 [+1.3]	17.1 [+2.3]	13.7 [−3.4]
CC outer	14.3	16.4 [+2.1]	17.5 [+3.2]	14.7 [−2.8]
CHOROID whole	14.8	15.9 [+1.1]	14.9 [+0.1]	13.4 [−1.5]
CHOROID inner	14.6	15.7 [+1.1]	14.3 [−0.3]	12.7 [−1.6]
CHOROID outer	16.1	17.4 [+1.3]	16.9 [+0.8]	15.1 [−1.8]

*p-value < 0.05 (Student’s *t*-test); OCT, optical coherence tomography; OCTA, optical coherence tomography angiography; GCA, giant cell arteritis; CRT, central retinal thickness; GCC, ganglion cell complex; RNFL, retinal nerve fiber layer; ONH, optic nerve head; RPCP, radial peripapillary capillary plexus; LC, lamina cribrosa; SCP, superficial capillary plexus; DCP, deep capillary plexus; ORCC, outer retina to choriocapillaris; CC, choriocapillaris.

Regarding the OCTA macular vessel density parameters, the SCP whole, inner, and outer were statistically lower in the AION group compared with the unaffected eyes of the AION group (differences: −2.6 [*p* = 0.041], −2.8 [*p* = 0.036], and −3.0 [*p* = 0.043], respectively). Other comparisons of OCTA parameters were not statistically significant.

In addition to the quantitative analysis of OCTA parameters, we performed a qualitative, morphological evaluation of OCTA scans in the group of GCA patients with AION in acute and chronic phases, without ocular involvement, and in the group of unaffected eyes of GCA patients with AION.

**Figure 3:**
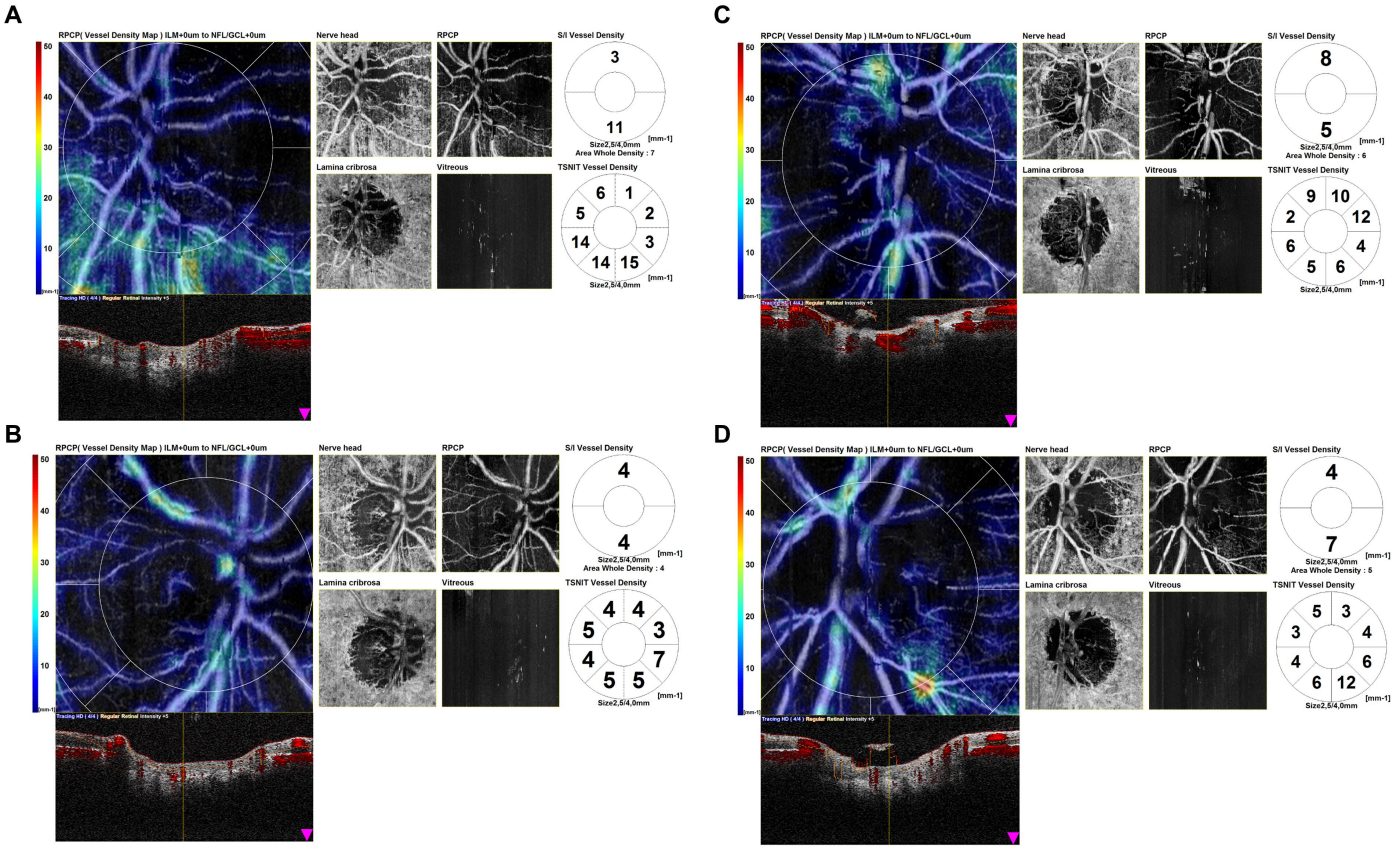
Optical coherence tomography angiography (OCTA) disk map scans of arteritic anterior ischemic optic neuropathy (AION) in the chronic phase. OCTA scans in eyes affected by AION in the chronic phase showed a profound vascular impairment. It is evident the reduction in vessel density in the peripapillary capillaries and the atrophy of the optic disk.

Eyes affected by AION in the chronic phase showed in OCTA scans a profound vascular impairment. Figure 3 shows four scans of the OCTA disk map of arteritic AION in the chronic phase. It is evident from the reduction in vessel density in the peripapillary capillaries and the atrophy of the optic disk.

**Figure 4:**
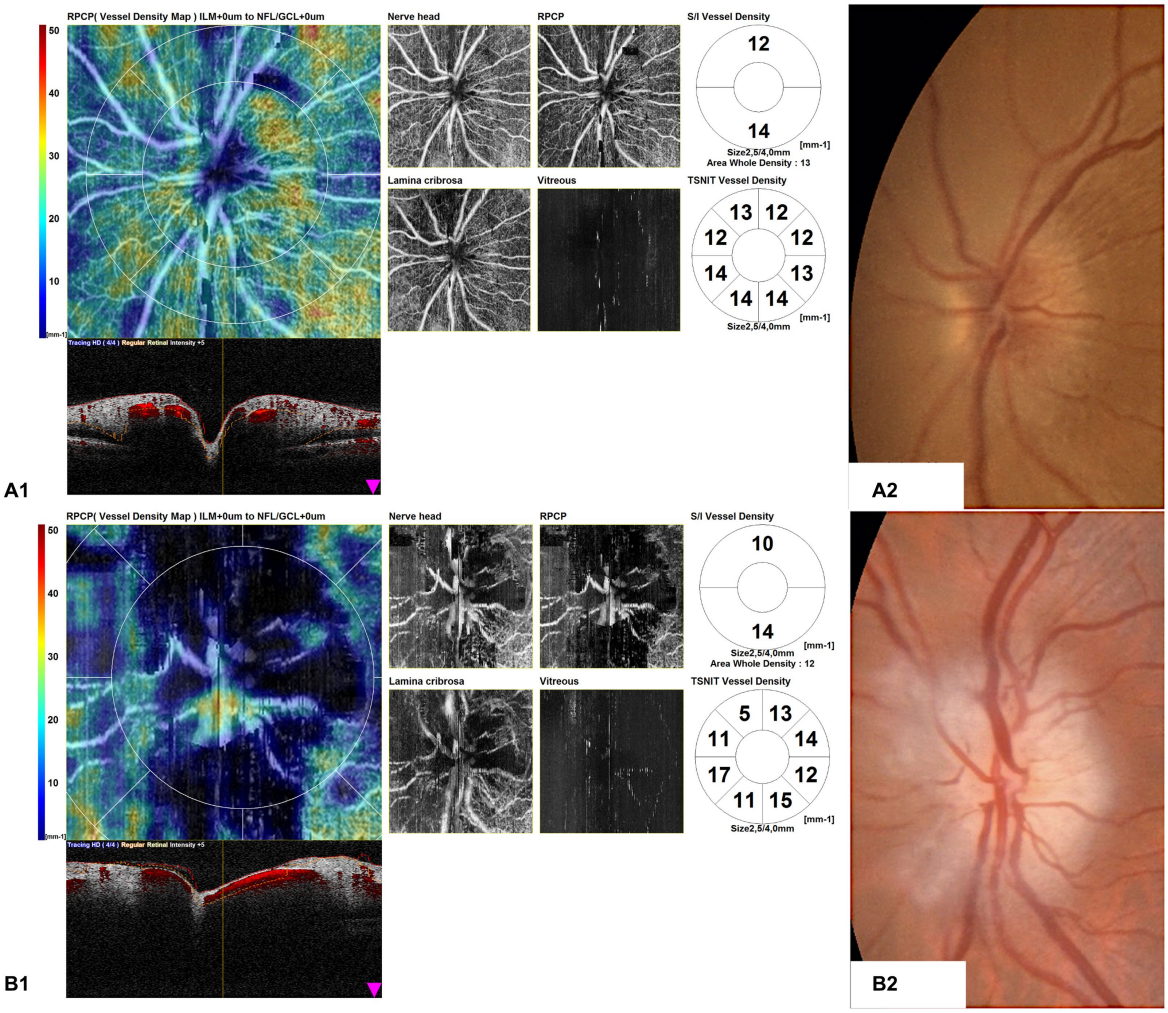
Optical coherence tomography angiography (OCTA) disk map scans of arteritic anterior ischemic optic neuropathy (AION) in the acute phase in two patients **(A1,B1)** and color fundus photographs of the same eyes **(A2,B2)**. **(A1)** shows the OCTA disk of a patient with AION in an early acute phase, performed 1 day after the visual symptom onset. Vascular tortuosity and telangiectasia are visible, but an important impairment of vessel density is not evident. **(B1)** shows the OCTA disk scan of an acute AION, performed after 4 days from the visual loss onset. The ischemia is more evident, and the peripapillary network is less visible around the edema area.

OCTA disk scans of two cases of arteritic AION in the acute phase are shown in Figure 4. Figure 4A1 shows the OCTA disk of a patient with AION in an early acute phase, performed 1 day after the visual symptoms’ onset. BCVA was 2/10 during the time of the OCTA scan. Vascular tortuosity and telangiectasia are visible, with only a slight decrease in peripapillary vascularity, but an important impairment of vessel density is not evident. Figure 4B1 shows the OCTA disk scan of an acute AION, performed after 4 days from the visual loss onset. BCVA was 1/20 during the time of the OCTA scan. The ischemia is more evident, and the peripapillary network is less visible around the edema area. There are some vascular drop-out zones, displayed by dark areas. The global aspect is the disappearance of the peripapillary regular vascular pattern.

**Figure 5:**
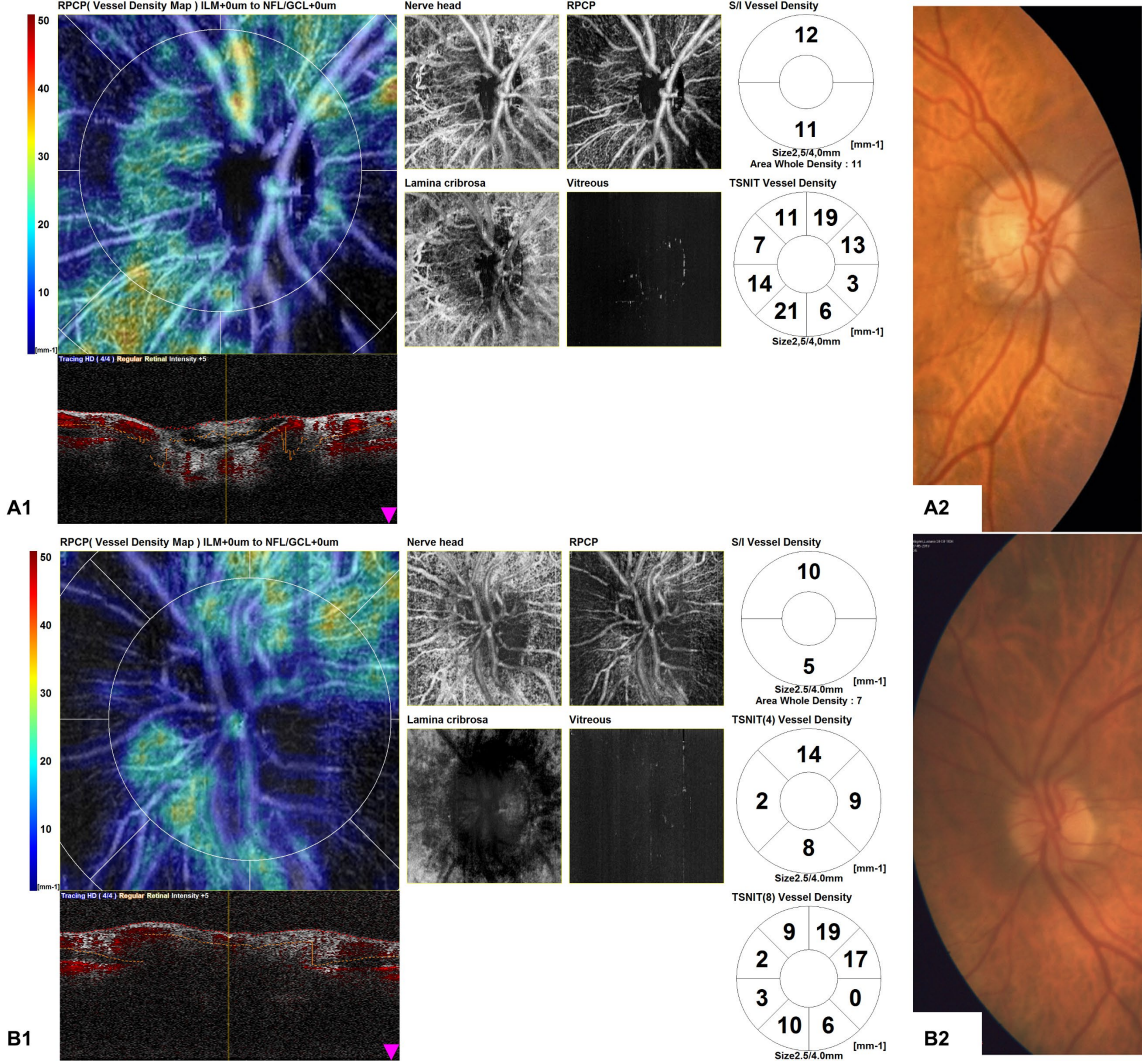
Optical coherence tomography angiography (OCTA) disk map scans of unaffected eyes of giant cell arteritis (GCA) patients with arteritic anterior ischemic optic neuropathy (AION) **(A1,B1)** and color fundus photographs of the same eyes **(A2,B2)**. The OCTA scans of unaffected eyes of GCA patients with AION showed areas of optic nerve hypoperfusion, with vessel density focal dropout.

Furthermore, the OCTA scans of unaffected eyes of GCA patients with AION showed areas of optic nerve hypoperfusion with vessel density focal dropout (Figure 5).

**Figure 6:**
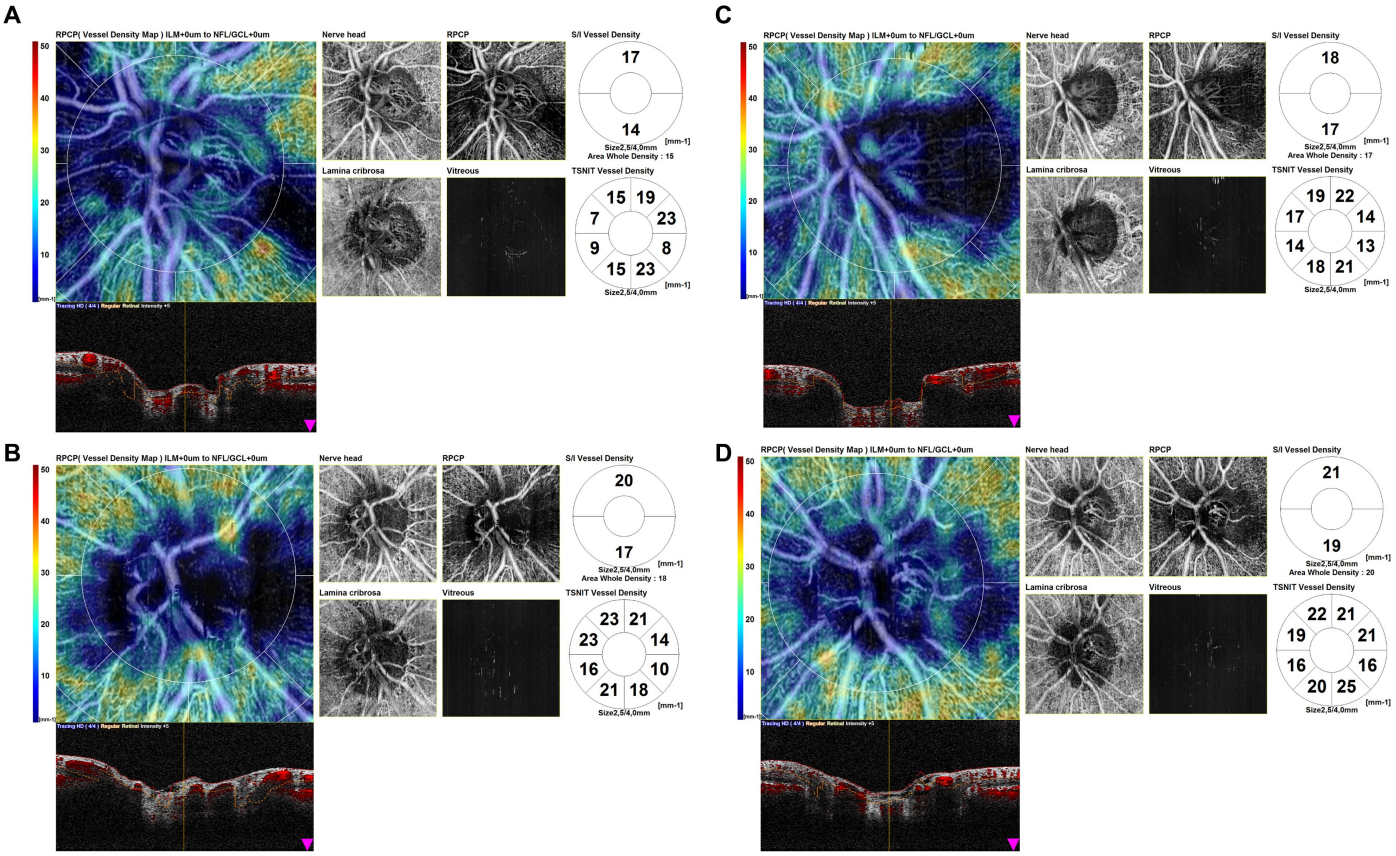
Optical coherence tomography angiography (OCTA) disk map scans of the eyes of giant cell arteritis (GCA) patients without ocular involvement. OCTA scans show patterns of localized hypoperfusion areas in patients with GCA without ocular involvement.

We also found patterns of localized hypoperfusion areas in patients with GCA without ocular involvement (Figure 6).

## Discussion

In our study, we conducted a quantitative and qualitative analysis of OCT and OCTA parameters in a subset of GCA patients with both acute and late-stage AION, in the affected and unaffected eyes, in a subset of GCA patients without ocular involvement, and in a group of healthy subjects. This analysis had the purpose of better characterizing the subtle perfusion features of the arteritic AION and to research some subclinical alterations in GCA patients without ocular involvement and in the unaffected eyes of patients with AION.

The OCTA analysis of the disk and the macula of eyes affected by arteritic AION has been described only in a limited number of studies (10–13). More OCTA studies have instead been conducted on the non-arteritic form (NAION) (15–21).

In our study, all of the OCTA disk variables analyzed were statistically lower in the group of patients with AION in the affected eye compared to the unaffected eye, similar to other studies conducted on arteritic AION and NAION that report a reduction in vessel density in impaired eyes compared to unaffected eyes (10–13, 15–21).

We may hypothesize that in AION in the acute phase, the combined influence of optic disk edema and ocular blood flow reduction might determine an overall perfusion reduction of the ONH, which may also cause irreversible vascular damage. The hypoperfusion of the radial peripapillary capillary network in the late stage may be a secondary change accompanying retinal ganglion cell loss and diminished metabolic needs, as found in studies on NAION (20). The retinal radial peripapillary capillary network is thought to nourish the RNFL in the peripapillary field (22). In AION, the primary site of the ONH lesion is mainly nourished by the microcirculation of the posterior ciliary artery, and the primary ischemia is not at the level of the radial peripapillary capillaries. Then the loss of peripapillary capillaries in AION might be due to secondary ischemic RNFL loss or to a reduction of metabolic demand, similar to findings in studies on NAION (23). It can be hypothesized that these vascular changes may induce RNFL loss by ischemia, with a consequent reduction of visual field sensibility. Furthermore, the choroidal perfusion seems to be impaired in AION, as shown by Pellegrini et al., who compared the choroidal vascularity index, defined as the ratio of luminal area to total choroid area, in patients with arteritic AION, NAION, and control subjects in a retrospective cross-sectional study. The authors found a significantly lower peripapillary and macular choroidal vascularity index in arteritic AION patients compared to both patients with NAION and control subjects (13).

In our study, the morphological analysis of OCTA disk maps showed in eyes affected by AION in the late phase a profound vascular impairment, with a visible reduction in vessel density in the peripapillary capillaries and a clear boundary of the ischemic area, while in eyes with acute AION it showed vascular tortuosity, with only a slight decrease in peripapillary vascularity. We may hypothesize that in AION, in the early acute phase, the blood flow may be affected mostly by mechanical compression of the capillaries, proportionally to the axonal swelling degree, while in the late stages, an overall perfusion reduction of the ONH may occur, leading to irreversible vascular damage.

In the group of GCA patients without ocular involvement, we interestingly found lower parameters of disk vessel density if compared to healthy controls, although statistically significant for only two parameters (ONH whole and ONH inferior). This quantitative difference was concordant with the morphological observation of focal hypoperfusion areas in the OCTA scans in GCA patients without ocular involvement and can be considered an interesting finding because it may indicate the presence of subclinical blood circulation impairment.

We also quantitatively assessed the OCTA parameters of unaffected eyes of patients with AION in comparison with the eyes of GCA patients without ocular involvement, and we found some parameters higher and others lower than the group of GCA patients without ocular involvement, without significant differences; only one parameter (ONH inferior) was statistically higher in the group of the unaffected eye. The qualitative analysis showed areas of focal hypoperfusion in OCTA scans and also in the group of unaffected eyes of GCA patients with AION. However, this result is in contrast with the study of Pierro et al. (12), which did not find vascular changes in the unaffected eyes of patients with AION in comparison with healthy controls.

Regarding the OCTA macular variables, the vessel densities were lower in the group of patients with AION in the affected eye in comparison with the unaffected eye, significantly affecting the SCP whole and the SCP inferior parameters. Similarly, in a Chinese study, the macular vessel density in patients affected by NAION in the acute, subacute, and chronic phases was analyzed in comparison with healthy controls. The authors found a significant reduction in the macular superficial vessel density of patients with NAION in acute, subacute, and chronic phases compared with healthy controls. In this study, the deep vessel density was not significantly reduced. They also found a decrease in superficial vessel density along with the course of the disease and demonstrated a correlation with structure and visual function (24). This result is in agreement with other findings in the literature, indicating that the superficial retinal layer seems to be more involved than the deeper choroid layer in the AION eyes (both arteritic and non-arteritic) (25).

According to our results, we may hypothesize that similarly to eyes with AION, a perfusion impairment involving the macula could occur in the acute stage due to disk edema and impairment in ganglion cells, and in the late stage due to the atrophy of the retinal nerve tissue leading to lower metabolic demand and finally resulting in decreased capillary perfusion, as described in NAION (26).

Regarding the structural OCT parameters, the macular CRT did not show significant variations in the different groups, while we found some significant differences in sectors of RNFL thickness. However, we did not consider these parameters clinically significant due to the analysis in the same group of both AION in the acute phase, in which the ONH was edematous, and AION in the chronic phase, in which atrophy was predominant. The GCC superior and inferior thicknesses were significantly lower in the AION group compared to the unaffected eyes of the AION group, and this result may reflect the damage of the retinal nerve tissue, mainly retinal ganglion cells and their axons, occurring after AION.

The present study has some limitations. First, the sample size was small, although GCA is a rare condition. Furthermore, we have to consider that GCA patients more frequently display cardiovascular risk factors, such as dyslipidemia and hypertension, than non-vasculitis patients (27), and this fact may have an impact on the results of our study.

The inclusion of the same sample of patients with AION both in the acute phase and in the late phase can represent another limitation because, generally, in the acute phase, the ONH is edematous, while in the late phase, it is atrophic. We analyzed these data together because it was difficult to make analyses separating the two groups due to the small size of the population studied, but that fact made the structural analysis of RNFL not clinically significant. It can be useful in further studies to analyze the chronic and acute phases of arteritic AION separately, increasing the size of the population. Furthermore, it could be interesting to analyze the RNFL defects in comparison with the OCTA values, as some authors did, finding a correlation between vessel density impairment and RNFL defects (12, 16, 17, 23, 28), and further analyze the correlation of the visual field with the OCTA parameters and perimetric alterations.

Moreover, we performed a cross-sectional analysis of perfusion characteristics revealed with OCTA, but a longitudinal study of patients analyzed in acute phase AION and during a follow-up could be useful to explore the changes in perfusion over time and the role of optic disk perfusion alterations in the pathogenesis of non-acute phase AION.

## Conclusion

In conclusion, OCTA can be considered a useful non-invasive tool that allows a quantitative and qualitative analysis of the vascular perfusion of the ONH. This imaging technique can be used to evaluate the vascular impairment in eyes with GCA-correlated visual loss but also to explore potential subclinical perfusion defects in the unaffected eyes and the eyes of GCA patients without ocular involvement. We found, as expected, a profound vascular impairment in eyes affected by AION, but it was newsworthy to find areas of hypoperfusion in the eyes of patients with GCA without ocular involvement that had good BCVA and were clinically unremarkable. We hypothesized that these areas of lower vessel density might represent areas of subclinical hypoperfusion that cannot be appreciated ophthalmoscopically.

We found perfusion defects in the eyes of patients with GCA without ocular involvement that were clinically unremarkable. Further studies, with more enrolled patients, are required to assess the role of OCTA in the characterization of vascular impairment in arteritic AION and the detection of subclinical features of low perfusion in GCA patients without a manifest ocular involvement.

## Data availability statement

The raw data supporting the conclusions of this article will be made available by the authors, without undue reservation.

## Ethics statement

The studies involving humans were approved by Careggi University Hospital Research Ethics Board. The studies were conducted in accordance with the local legislation and institutional requirements. The participants provided their written informed consent to participate in this study.

## Author contributions

LV: Writing – original draft, Writing – review & editing, Conceptualization, Data curation, Investigation, Methodology, Project administration. CN: Writing – original draft, Writing – review & editing, Conceptualization, Data curation, Investigation, Methodology, Project administration. GiuV: Writing – original draft, Writing – review & editing, Conceptualization, Data curation, Formal analysis, Investigation, Methodology, Validation, Visualization. DB: Writing – review & editing, Conceptualization, Data curation, Methodology, Supervision, Visualization. DG: Writing – original draft, Writing – review & editing, Data curation, Methodology, Validation, Visualization. MU: Writing – original draft, Conceptualization, Data curation, Funding acquisition, Investigation, Methodology, Project administration. AP: Writing – review & editing, Methodology, Validation, Visualization. DM: Writing – review & editing, Investigation, Visualization. FB: Writing – review & editing, Investigation, Visualization. GiaV: Writing – review & editing, Data curation, Formal analysis, Resources, Software, Visualization. FG: Writing – review & editing, Writing – original draft, Funding acquisition, Project administration, Resources, Supervision, Visualization.
